# Management and outcome of pelvic fracture associated with vaginal injuries: a retrospective study of 25 cases

**DOI:** 10.1186/s12891-019-2839-y

**Published:** 2019-10-22

**Authors:** Pengyu Li, Dongsheng Zhou, Baisheng Fu, Wenhao Song, Jinlei Dong

**Affiliations:** Department of Orthopedic Surgery, Shandong Provincial Hospital Affiliated to Shandong University, 324 Jingwu Road, Jinan, Shandong China

**Keywords:** Pelvic fracture, Vaginal injury, Clinical outcome

## Abstract

**Background:**

Pelvic ring fractures associated with vaginal injuries were rarely reported due to low incidence. The displaced segments of pelvic ring may increase the risk of vaginal injury. The aim of this retrospective study was to evaluate the correlation between pelvic fracture and vaginal injury.

**Methods:**

We conducted a retrospective review of 25 patients with pelvic fractures associated with vaginal injury treated at our institution. The medical records of these patients were collected and 24 patients were followed-up for 10–36 months.

**Results:**

All patients suffered anterior pelvic ring fracture. Young-Burgess fracture classification and compromised pubic symphysis were related to severity of vaginal injury. Gauze packing was done in 6 patients and 18 patients received surgical repair. Infection occurred in 6 patients, among them 4 were due to delayed diagnosis. Factors associated with pelvic outcome were age, urethral injury, and infection. Four patients suffered pain in sexual intercourse but no influence factor found correlated to sexual function.

**Conclusion:**

VS type pelvic fracture and compromised pubic symphysis were related to higher severity of vaginal injury. Disruption of anterior ring and an unstable pelvic ring caused by forces on coronary and axial plane may increase the risk of vaginal injury.

**Trial registration:**

ChiCTR1900020540. Registered 28 January 2019. Retrospectively registered. Trial registry: Chinese Clinical Trial Registry.

## Background

Pelvic fractures are often caused by high energy trauma with high mortality and the incidence reported is from 8 to 15% [1, 2]. Urogenital and lower intestinal tracts are tend to be injured by displaced pelvic ring [3–5]. Vaginal laceration after pelvic trauma is rare with an incidence about 2 to 4% in previous reports [6–9]. The reduced bleeding because of the contraction of vaginal smooth muscles which acts as a post-traumatic stress reaction and the severe injuries of other organs associated with pelvic fracture may result in missed diagnosis of vaginal injury and potentially lead to various complications including pelvic infection, vaginal stenosis and sexual dysfunction [6, 10]. It might be considered as an underestimated issue in the care of high energy blunt pelvic trauma in female patients. However, studies of pelvic fractures associated with vaginal injuries are rare and most are case reports which mainly present treatment process and clinical outcomes [11–14] but lack of a systemic study. Considering the anatomical relationship, fractured segments of pelvic ring may increase the risk of vaginal injury. We hypothesized that fracture pattern may have influence on the severity of vaginal injury. Therefore, we reviewed patients treated in our institute with an aim to evaluate the relationship between pelvic fracture and vaginal injury.

## Methods

From January 2004 to April 2017, 341 female patients with pelvic fractures were treated at our institution. The inclusion criterion of this study was female patients with pelvic fractures associated with vaginal injuries and 27 patients were identified. The incidence of vaginal injury after pelvic trauma was 7.9% according to our data. Exclusion criterion was patients with penetrating injury and 2 patients were excluded. Totally 25 patients were enrolled in this study. Permission for this study was obtained from the authors’ institution. Informed consent was obtained from all individual participants.

### Patients’ details

The data gathered from each patient included: age, type of pelvic fracture, mechanism of injury, degree of vaginal injury, associated anorectal and urethral injury, orthopedic management of pelvic fracture, Injury Severity Score (ISS) [15].

### Classification of pelvic fractures and vaginal injury

Relationship between pelvic fracture and vaginal injury was our most concern so we used different classifications of pelvic fracture including Tile [16] system, Young-Burgess classification [17] and with and without compromised symphysis. The vaginal injury was classified using the “Injury Scoring Scaling” of the American Association for the Surgery of Trauma [18]: first degree means I and II grade injury including contusion, hematoma and superficial laceration (mucosa only); second degree means III grade including laceration which deep into fat or muscle and third degree means IV and V grade degree including laceration into cervix or peritoneum or injury into adjacent organs.

### Outcome evaluation

The functional outcome was evaluated using Majeed scoring system [19] and the residual displacement was assessed according to the method of Tornetta and Matta [20].

### Statistical analysis

Statistical analysis was done using SPSS version 16.0 for windows (SPSS Inc., Chicago, Illinois). The non-parametric Kruskal-Wallis test was used to assess the relationship between fracture patterns and degree of vaginal injury and to identify factors which were associated with clinical outcome. The Fisher exact test was used to identify factors which were associated with sexual function. A *p* value of 0.05 or less was considered to be statistically significant.

## Results

### Patients’ details

Twenty-five pelvic fracture patients with vaginal injury met the inclusion criterion during the study period. The mean age was 32.8 ± 12.4 years old (range, 17–55). The mean ISS was 31.4 ± 14.9 (range, 10–57). Nine patients in this series had an ISS of greater than 25. The mean time from injury to emergency department was 2.02 ± 0.8 h (range, 1–3.5). Most injuries were caused by traffic accidents (22 patients, 88%) while 2 patients falling from height and one patient encountered with crush injury.

### Pattern of pelvic fractures and vaginal injury

Six patients (24%) with mucosal injury were categorized as first degree. Twelve patients (48%) suffered second degree injury, presented with involvement of vaginal muscularis but not penetrating through rectum or urethra. Seven patients (28%) suffered third degree injury. Four of them suffered vaginal-rectal penetrating injury and vaginal-urethral penetrating wound was presented in 3 patients. All diagnoses were made by a gynecologist and vaginal speculum was used when necessary.

According to Tile classification, the most common injury pattern was type B fracture, occurring in 18 patients (72%). Another 7 patients (28%) sustained a type C fracture, and none had type A fracture. The severity of vaginal injury didn’t differ between type B and C fractures according to our classification (*p* = 0.208, Table 1). In Young-Burgess system, 20 patients sustained anterior-posterior compression (APC), five patients sustained vertical shear (VS) injuries and no one showed lateral compression (LC) injuries. The VS fracture patients showed higher severity of vaginal injury compared to patients with APC fracture (*p* = 0.034, Table 1).

**Table 1:** Facture pattern and vaginal injury

Type	Subtype	Vaginal injury				*P* value
		1	2	3	Total	
Tile	A	0	0	0	0	0.208
	B	5	10	3	18	
	C	1	3	3	7	
Young-Burgess	APC	6	11	3	20	0.034*
	VS	0	2	3	5	
	LC	0	0	0	0	
Compromised pubic symphysis	Yes	1	7	5	13	0.024*
	No	5	6	1	12	

*Statistically significant *P* value

All patients suffered disruption of anterior pelvic ring. Most patients suffered pubic ramus fracture (20 patients, 80%). Among them, 10 patients presented unilateral fracture and 10 presented bilateral fracture. Four patients were also combined with pubic symphysis separation. Thirteen patients had a compromised pubic symphysis including 9 patients with pubic symphysis separation and 4 patients presented a rather rare floating pubic symphysis which means fracture of the bilateral superior and inferior pubic rami and ischial rami. A compromised pubic symphysis was related to more severe vaginal injury. (*p* = 0.024, Table 1).

### Treatment of pelvic fracture

Twenty-four patients survived initial resuscitation phase. External fixation was performed for 11 patients and 5 patients didn’t receive further surgery. Fourteen patients had open reduction and internal fixation (ORIF), among them first-stage ORIF was only performed for 5 patients. The other 9 patients had second-stage or delayed surgery due to unstable hemodynamic condition and abscess formation (7–14 days). In these 9 patients, external fixation was applied in 6 patients at first stage and pelvic binder was used for 2 patients. Conservative treatment was done in the other 5 patients.

### Treatment of vaginal injury

No special treatment but only gauze packing was done in the 6 patients with mucosal injury and one patient with second degree injury. All the other patients with second or third degree injury went through surgical repair. Fourteen patients had primary closure and 4 patients had secondary vaginal repair. The wound was sutured through interrupted suture by a gynecologist with 1–0 absorbable sutures.

### Treatment of associated injury

Anorectal injury occurred in 15 patients, all of them were performed colostomy except for one patient died in emergency room. Another patient with severe vaginal-perineal laceration but no injury of rectum also had protective colostomy. All these patients were performed primary debridement and colostomy with anal sphincter repaired and distal lumen rinsed to avoid secondary contamination. Fifteen patients were complicated with urinary tract injuries. Of these 3 were bladder injuries. One patient acquired laparotomy with surgical repair of the bladder due to serious damage. Two patients had slight damage and were treated with indwelling catheter. Twelve patients suffered urethral disruption. Six of them required primary suprapubic drainage and subsequent delayed repair. Two patients with vaginal-urethral penetration had urethral realignment. The others were treated conservatively.

### Outcome evaluation

Totally 24 patients were followed up at mean 17.7 months (range, 10–36). The function evaluation was done at least 12 months after discharge. No patient showed signs of radiographic nonunion. The average time of fracture healing was 4.3 ± 1.2 months (range 3–7 months). The radiologic outcome was assessed using Tornetta and Matta criteria. Only 2 patients showed unsatisfactory results. One was fair and one was poor. Factors associated with pelvic outcomes were shown in Table 2. Eighteen patients were assessed as excellent, four patients were good, two patients were fair and none was poor. Pelvic outcome was better significantly among younger patients (*p* = 0.043) and patients without urethral injury (*p* = 0.02). Infected patients showed worse results (*p* = 0.005). Though management of pelvic fractures has insignificant influence on pelvic function (*p* = 0.055), patients received external fixation in isolation seemed to present worse pelvic function.

**Table 2:** Factors affecting pelvic outcome

Group	Subgroup	Pelvic outcome					*P* value
		Excellent	Good	Fair	Poor	Total	
Age	≤30	14	0	1	0	15	0.043*
	>30	4	4	1	0	9	
ISS	≥25	8	1	0	0	9	0.208
	<25	10	3	2	0	15	
Tile classification	A	0	0	0	0	0	0.510
	B	14	2	2	0	18	
	C	4	2	0	0	6	
Young-Burgess classification	LC	0	0	0	0	0	0.913
	APC	15	3	2	0	20	
	VS	3	1	0	0	4	
Compromised Pubic symphysis	Yes	9	3	0	0	12	0.515
	No	9	1	2	0	12	
Vaginal injury	1st degree	6	0	0	0	6	0.284
	2nd degree	8	3	1	0	12	
	3rd degree	4	1	1	0	6	
Vaginal repairing	Gauze packing	6	0	0	0	6	0.113
	Surgical repair	12	4	2	0	18	
Fixation technique Infection	Non-operative	5	0	0	0	5	0.055
	Isolated external fixation	2	1	2	0	5	
	ORIF Yes No	11 2 16	3 2 2	0 2 0	0 0 0	14 6 18	0.005*
Urinary tract injury	Yes	8	4	2	0	14	0.020*
	No	10	0	0	0	10	
Anorectal injury	Yes	9	3	2	0	14	0.142
	No	9	1	0	0	10	

*Statistically significant *P* value

*ISS* Injury Severity Score, *ORIF* Open reduction and fixation

Four patients complained of pain in sex intercourse at last follow-up but did not demand further treatment. Nine patients were still virgin at the last follow-up. Sexual function was assessed in the other 15 patients using Fisher exact test but no significant factor related to sexual pain was found (Table 3). All surviving patients had normal menstruation while one patient in menopause at last follow-up. Nine patients had at least one child delivered and one patient was pregnant at the time of the latest follow-up. Six patients were performed cesarean section and three patients had natural birth.

**Table 3:** Factors affecting sexual function

Group	Subgroup	Pain in sexual intercourse					*P* value
		No		Yes		Total	
Age	≤30	5		3		8	0.569
	>30	6		1		7	
ISS	≥25	5		1		6	0.604
	<25	6		3		9	
Tile classification	A	0		0		0	0.999
	B	7		3		10	
	C	4		1		5	
Young-Burgess classification	LC	0		0		0	0.999
	APC	9		3		12	
	VS	2		1		3	
Compromised pubic symphysis	Yes	6		1		7	0.569
	No	5		3		8	
Vaginal injury	1st degree	2		1		3	0.590
	2nd degree	5		3		8	
	3rd degree	4		0		4	
Vaginal repairing	Gauze packing	2		1		3	0.736
	Surgical repair	8		2		10	
Fixation technique Infection	Non-operative	1		1		2	0.110
	Isolated external fixation	1		2		3	
	ORIF Yes No	9 3 8		1 2 2		10 5 10	0.560
Urinary tract injury	Yes	7		3		10	0.999
	No	4		1		5	
Anorectal injury	Yes	7		4		11	0.516
	No	4		0		4	

*ISS* Injury Severity Score, *ORIF* Open reduction and fixation

### Complications

Infection occurred in 6 patients. Four patients developed pelvic abscess and were treated with abscess incision and drainage. Other two patients were treated with debridement and changing dressings. Sensitive antibiotic drugs were chosen for infected patients based on the drug sensitivity tests. Vaginal stenosis occurred in 2 patients. No patient showed associated vessel or nerve injuries. Mechanical complications like instrumentation failure did not occur.

### Limitations

There were several limitations in this study. The sample size was comparatively small and there was probably high number of undetected vaginal injuries. It was a retrospective study with long time span and long-termfollow-ups were not able to acquire from all patients. This study also lacked a more concrete evaluation of sexual function.

## Discussion

Our hypothesis that fracture pattern may have influence on vaginal injury was proven by the result that the severity of vaginal injury was correlated with Young-Burgess fracture classification and the presence of compromised pubic symphysis. Our results showed that VS type fractures and compromised pubic symphysis purported more severe vaginal injury (*p* < 0.05, Table 1). All injuries were caused by blunt forces and no penetrating wound by external sharp objects so the displaced fracture segments had important influence on vaginal injury. Niemi and Norton depicted that the vaginal wall may be pulled, penetrated by the fracture ends of pelvic anterior ring as well as separated or floating pubic symphysis or pressed by the decreased pelvic volume [6]. Fowler et al. also emphasized that pubis and ischium could bruise or penetrate vaginal wall [13]. We found this injury occurred more often in certain kinds of pelvic fractures. As the disruption of anterior pelvic ring was seen in all patients, it could be a risk factor of vaginal injury. According to Tile classification, all patients were B and C type. Though no difference was shown between 2 groups, this result showed that an unstable pelvic ring probably increased the risk of vaginal injury. Young-Burgess classification is based on mechanism of injury. Considering that LC type fracture was not seen in these patients, we think vagina is more easy to get hurt when pelvis is encountered with force on coronary or axial plane.

Function recovery is important in patients with vaginal injury for the high incidence of vaginal stenosis and sexual dysfunction [6, 10, 13, 21]. Harvey-Kelly et al. reported that rate of sexual dysfunction after pelvic fracture could be as high as 40% [22]. Vallier’s study showed 25.7% female pelvic fracture patients reported pain in sexual intercourse [8]. In 15 patients with sexual experience, four patients complained of pain in sex. One patient suffered mucosal injury and two presented with second degree injury. The rest one patient suffered vaginal-urethral penetration with developed pelvic abscess and accepted vaginal repair at second stage. Due to small case series, no influence factor was found correlated to sexual function. Spontaneous conception and normal vaginal delivery are possible after pelvic fracture [12], the result from this study supported this conclusion. Twenty-three patients of survived 24 patients were women of childbearing age, nine patients had conception and delivered successfully. Six patients were performed cesarean section and three patients had natural birth, the rate was higher than average rate over the world. We think that this phenomenon is due to the condition of obstetrics in China. For patients with minor damage in vagina, simply gauze packing was sufficient for hemostasis and capable of preventing vaginal adhesion and narrow. No significant relationship was found between degree or treatment of vaginal injury and pelvic outcome.

Vaginal injury may increase the risk of infection especially when diagnosed not in time [6]. Infection was the most common complication and significantly associated with bad pelvic outcome (*p* < 0.05, Table 2) which occurred in 6 patients. Four patients were transferred from local hospital and developed pelvic abscess on admission. The diagnoses of vaginal laceration were delayed 30, 36, 36 and 48 h. Vagina has rich blood supply but the contraction of smooth muscle due to post-traumatic stress response may lead to insignificant bleeding. No obvious symptom on admission may lead to the omission of diagnosis. Long-term complications of delayed diagnosis include vaginal stenosis, atresia and urethra- or recto-vaginal fistula [10, 23]. Vaginal stenosis occurred in two patients with delayed diagnosis but neither of them received further treatment at the last follow-up. We reviewed previous articles and found that the main reason of missed diagnosis was lack of thorough examination [23]. Since delayed diagnosis may lead to increased risk of infection and late complications, a thorough vaginal examination should be performed by a senior gynecologist in a trauma center when patients come with high risk of vaginal injury.

The selection of fixation technique in open pelvic fractures remains controversial. We previously reported that the clinical outcomes were better in the operative group than non-operative group by Majeed score in open pelvic fractures [24]. In present study, five patients with pelvic fracture were treated non-operatively and all achieved excellent outcome. For 11 patients with unstable hemodynamics or infection, external fixation was used to acquire temporal stabilization and hemostasis through limiting pelvic volume. Six patients received further open reduction and internal fixation at second stage and showed satisfactory results. Five patients had only external fixation. All 5 patients sustained Tile B and APC fractures. These patients had persistent infection or poor skin integrity. They had long hospital stay and a stable pelvis was shown radiologically. External fixation was used as definitive fixation in these patients. Traditionally, only external fixation technique is used in open pelvic fracture patients for a lower risk of infection [25]. But compared with internal fixation technique, it may provide limited stability for pelvic injuries [26]. Our previous experiences were in favor of using internal fixation in those unstable pelvic fractures without gross contamination in the fracture region [27]. This study showed that though statistically insignificant (*p* = 0.055), patients received external fixation in isolation tended to have relative bad pelvic outcome. We recommend internal fixation if patients’ conditions permit.

## Conclusions

Here we propose a new finding that VS type pelvic fracture and compromise pubic symphysis are related to higher severity of vaginal injury. Disruption of anterior ring and an unstable pelvic ring caused by forces on coronary and axial plane may increase the risk of vaginal injury. However, no factor found related to sexual dysfunction and more cases need to be analyzed in future study.

## Data Availability

The data used in the current study are available from the corresponding author on reasonable request.

## Abbreviations

APC: Anterior-posterior compression
ISS: Injury severity score
LC: Lateral compression
ORIF: Open reduction and internal fixation
VS: Vertical shear
